# Strategic applications of methylene thioacetal bonds as disulfide surrogates in peptide drug discovery

**DOI:** 10.3389/fchem.2025.1637329

**Published:** 2025-06-27

**Authors:** Yaqi Zhou, Dongyuan Wang, Jiean Xu, Nan Zheng

**Affiliations:** ^1^ Department of Physiology and Research Centre of Basic Integrative Medicine, School of Basic Medical Sciences, Guangzhou University of Chinese Medicine, Guangzhou, China; ^2^ Department of Pharmacy, Union Hospital, Tongji Medical College, Huazhong University of Science and Technology, Wuhan, China; ^3^ Translational Innovation Center, Shenzhen Bay Laboratory, Shenzhen, China

**Keywords:** disulfide surrogate, methylene thioacetal bond, stability and bioactivity, peptide drug discovery, peptide synthesis

## Abstract

Disulfide bonds are indispensable structural motifs in bioactive peptides, stabilizing conformations which are critical for molecular recognition and biological activity. However, their intrinsic chemical lability under physiological and manufacturing conditions has long presented challenges in peptide drug development. Efforts to address these limitations have yielded a diverse array of disulfide bond surrogates, each with distinct advantages and constraints. Among these, methylene thioacetal linkages have recently emerged as a particularly promising method offering a favorable balance of structural fidelity, synthetic accessibility, and chemical stability. This review summarizes the biological importance and limitations of native disulfide bonds, surveys established strategies for disulfide bond mimicry, and provide a comprehensive summary of research leveraging methylene thioacetal chemistry as an emerging tool in the design of next-generation peptide therapeutics.

## 1 Introduction

Peptide-based therapeutics have emerged as a rapidly expanding class of drug candidates, offering high target specificity, favorable pharmacokinetic properties and unique ability to modulate complex biological targets (Wang et al., 2022). Advances in peptide synthesis technologies, including solid-phase peptide synthesis (SPPS) and liquid-phase peptide synthesis (LPPS), as well as the emerging field of green and sustainable techniques, have enabled the efficient design and production of complex and challenging peptide targets (Ferrazzano et al., 2022; Stefanucci et al., 2025). Recent advances in synthetic methodology, formulation technologies, and delivery systems have revitalized the interest in therapeutic peptides, prospering their clinical relevance across oncology, endocrinology, infectious diseases, and immunotherapy (Anand et al., 2023). Distinguished by their conformationally constrained structures and potent, often exquisitely selective biological activities, disulfide-containing peptides represent a highly privileged class of bioactive molecules which continues to inspire innovative approaches to therapeutic development (Hogg, 2003). These structural features, conferred by disulfide bonds, endow the peptides with the ability to engage challenging biological targets (Erak et al., 2018). Therefore, a versatile class of disulfide-containing peptides, such as hormones and toxins, has been widely explored as valuable pharmacophore templates and starting points in the development of peptide therapeutics (Tyler et al., 2023). Beyond nature’s repertoire, advances in combinatorial and display technologies, such as phage, mRNA, and yeast display, have enabled the generation of artificial disulfide-containing peptides and mini-proteins with tailored binding specificities and improved pharmacokinetic profiles (You et al., 2024). These engineered scaffolds harness the structural advantages of disulfide bonds and expand chemical space beyond linear peptides (Li et al., 2022). Most recently, the artificial intelligence (AI)- or machine learning-driven rational design has emerged as another powerful tool in peptide therapeutics, enabling the *de novo* generation and optimization of disulfide-containing peptides (Ye et al., 2023).

As the therapeutic landscape for peptides continues to evolve, improving the chemical and metabolic stability of disulfide-containing peptides has emerged as a critical priority in peptide drug development (Al Musaimi et al., 2022). Accordingly, the development of novel, broadly applicable and cost-effective peptide modification methodology has become a central focus in the field (Gori et al., 2017). This review aims to provide a comprehensive framework for understanding the opportunities, challenges and current art associated with disulfide bond surrogate. Particular emphasis is placed on methylene thioacetal linkage which offer exceptional chemical stability, redox inertness and conformational control. Through selected case studies, we highlight the versatility and broad applicability of methylene thioacetal linkage in stabilizing disulfide-containing peptides, positioning it as a compelling platform for next-generation peptide therapeutics.

## 2 The function and limitations of native disulfides in bioactive peptides

**FIGURE 1:**
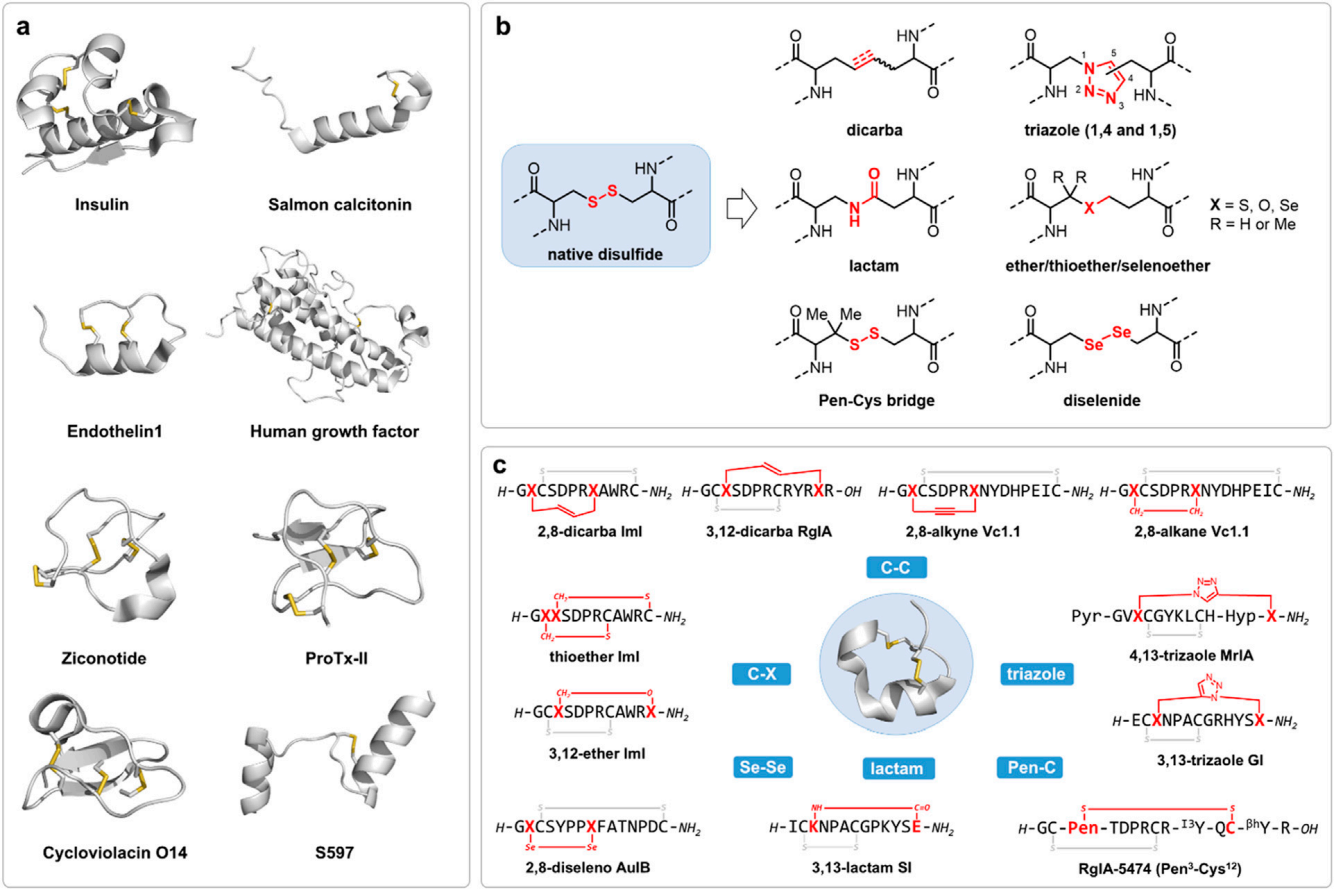
**(a)** Representative bioactive disulfide-containing peptides. The peptide backbones were represented in cartoon colored in grey and disulfide bonds were represented in stick colored in yellow. Structures generated from PDBs (insulin 1EVR; salmon Calcitonin 7TYN; endothelin1 8XVH; human growth factor 1HGU; ziconotide 7MIX; ProTx2 6N4R; Cycloviolacin O14 2GJ0; S597 8DTL. **(b)** The development of disulfide bond surrogates and **(c)** their application in disulfide-rich peptide compounds, exemplified by conotoxins.

A defining feature of many bioactive peptides is the formation of disulfide bond, a covalent linkage formed between the thiol groups of two cysteine residues (Cys) (Narayan, 2012). These disulfide-containing molecules, spanning native hormones (insulin, salmon calcitonin, human growth factor), venom toxins (ziconotide, spider toxin ProTx2), and plant derived cysteine knot mini-proteins (cyclotides), represent a structurally and functionally diverse class of peptides and mini-proteins exhibiting distinct biological activities, which are often regarded as privileged scaffolds in peptide drug discovery (Figure 1a) (Tyler et al., 2023; Mollica et al., 2014). Several have advanced directly to clinical application, highlighting their substantial therapeutic value and scientific significance (Dang and Sussmuth, 2017).

Disulfide bonds play a central role in stabilizing the three-dimensional structures by constraining conformational flexibility, thereby preserving bioactive peptide conformations essential for molecular recognition, receptor binding, and enzymatic regulation (Fass, 2012). In peptides lacking extensive hydrophobic cores, disulfide bridges are particularly vital in conferring resistance to thermal denaturation and proteolytic degradation. By dictating the precise spatial arrangement of pharmacophoric residues, disulfide linkages enable high-affinity and selective interactions with biological targets (Nagahara, 2011). Moreover, in certain physiological contexts, disulfide bonds serve as redox-sensitive molecular switches, thereby modulating peptide activity in response to changes in cellular oxidative stress or redox signaling (Chiu and Hogg, 2019).

Despite their structural and functional advantages, disulfide-containing peptides face notable limitations that restrict their broader therapeutic applications (Fass, 2012). The inherent redox sensitivity of disulfide bonds renders these peptides vulnerable to reduction in the intracellular environment, leading to premature cleavage and substantial loss of structural integrity and corresponding bioactivity. Disulfide bond scrambling, particularly during peptide synthesis, folding or *in vivo* circulation, can generate heterogeneous mixtures of isomers with variable or undesired biological activity. In peptides with multiple disulfide bonds, the combinatorial increase in possible regioisomeric arrangements further complicates the synthesis and folding, posing significant challenges for follow-up therapeutic development (Akondi et al., 2014). Furthermore, disulfide-containing peptides often exhibit limited metabolic stability due to susceptibility to enzymatic degradation by thiol-disulfide exchange reactions. Collectively, these drawbacks have spurred efforts to develop chemically robust, redox-stable surrogates and alternative covalent constraints that preserve or enhance the conformational and functional properties conferred by native disulfide bridges.

## 3 Reported disulfide surrogates in peptide modification

To address the limitations of native disulfide bonds, a broad array of chemical surrogates has been developed, each designed to emulate the structural and conformational constraints imposed by disulfide linkages while providing superior stability under physiological and manufacturing conditions (Figures 1b,c) (Gori et al., 2017).

Dicarba bonds, comprising alkyne, olefin or saturated hydrocarbon linkages, are among the most studied disulfide surrogates (Walensky and Bird, 2014). These chemically non-reducible all-carbon bonds, typically introduced via ring-closing metathesis (RCM) or ring-closing alkyne metathesis (RCAM), serve as effective surrogates for labile disulfide bonds, enhancing both the structural stability and metabolic resilience of peptide therapeutics (Blackwell and Grubbs, 1998). However, although several reported successful applications of dicarba analogues that resulted in improved stability while maintaining the original activity profile, their altered electronic and steric properties may occasionally perturb native-like peptide conformations, leading to reduced bioactivities (Stymiest et al., 2005; Hossain et al., 2009; MacRaild et al., 2009; Ma et al., 2024; Chhabra et al., 2014; Martin-Gago et al., 2014; Chen et al., 2023; Belgi et al., 2021).

Triazole linkages, generated through Cu(I) or Ru(I)-catalyzed azide-alkyne “click” cycloaddition, offer remarkable metabolic stability and synthetic versatility (Angell and Burgess, 2007). Nevertheless, the rigid, planar nature of the triazole moiety may introduce conformational constraints distinct from those imposed by native disulfide bonds, requiring careful optimization in structure-activity relationship studies (White et al., 2020; Williams et al., 2015; Gori et al., 2015; Knuhtsen et al., 2019; Tomassi et al., 2020).

Thioether bridges, formed via alkylation or native chemical ligation, introduce a non-reducible C-S bond that generally preserves native peptide topology (Cui et al., 2021). Although relatively straightforward to install, thioether linkages possess subtle differences in bond length and polarity that can influence peptide folding and biological activity (Dekan et al., 2011). Besides, thioether bonds replacement may lead to reduced hydrophobicity compared with native disulfide bonds. Notably, ether and selenoether bridges are more similar in structure and reactivity with the native disulfides and are oxidation resistant compared with thioether bridges (Zhao et al., 2020; Cui et al., 2021; de Araujo et al., 2014). Recently, a diaminodiacid (DADA) strategy was developed for streamlined and efficient synthesis of thioether replaced analogues (Cui et al., 2013; Zhao et al., 2023a; Zhao et al., 2023b).

Lactam bridges, created through amide bond formation between amine side chains (lysine, ornithine) and acid side chains (glutamic acid, aspartic acid), offering a highly efficient means of stabilizing cyclic or constrained peptides (Houston et al., 1996; Hargittai et al., 2000; Xu et al., 2024; Trotta et al., 2024). However, replacing a disulfide with an amide linkage may alter local hydrogen bonding networks and affect peptide dynamics.

The cysteine-penicillamine (Cys-Pen) bridges introduce steric hindrance via gem-dimethyl substituents on penicillamine, thus enhancing reductive stability while preserving native disulfide bond geometry (Hunt et al., 1993; Gajewiak et al., 2021; Li et al., 2024a; Di Maro et al., 2017). Despite these advantages, their application is constrained by synthetic complexity and potential steric effects on peptide conformation.

Diselenide bonds (Se-Se), formed by substituting cysteine residues with selenocysteine (Sec), have emerged as promising disulfide surrogates due to their unique redox properties and structural similarity to disulfide bonds (Mousa et al., 2017; Muttenthaler et al., 2010). The lower bond dissociation energy and redox potential of diselenide bonds facilitate more efficient oxidative folding pathways, enhancing the foldability and stability of peptides and proteins. For instance, replacing an internal disulfide bridge with a diselenide in human insulin significantly improved its folding efficiency and thermodynamic stability without compromising receptor binding affinity (Arai et al., 2023; Arai et al., 2017).

Besides the above-mentioned technologies, other methods constraining target peptides, or referred as peptide stapling techniques by cysteine or lysing conjugations, have provided alternative means for peptide drug designs (Li et al., 2024a; Stefanucci et al., 2017). While these surrogate strategies have advanced peptide drug discovery, many involve trade-offs in synthetic accessibility, conformational fidelity, or pharmacological performance. As such, the search for chemically robust, synthetically tractable, and biologically compatible alternatives remains a central pursuit in peptide therapeutics research.

## 4 Methylene thioacetal as disulfide surrogate in peptide therapeutics

**FIGURE 2:**
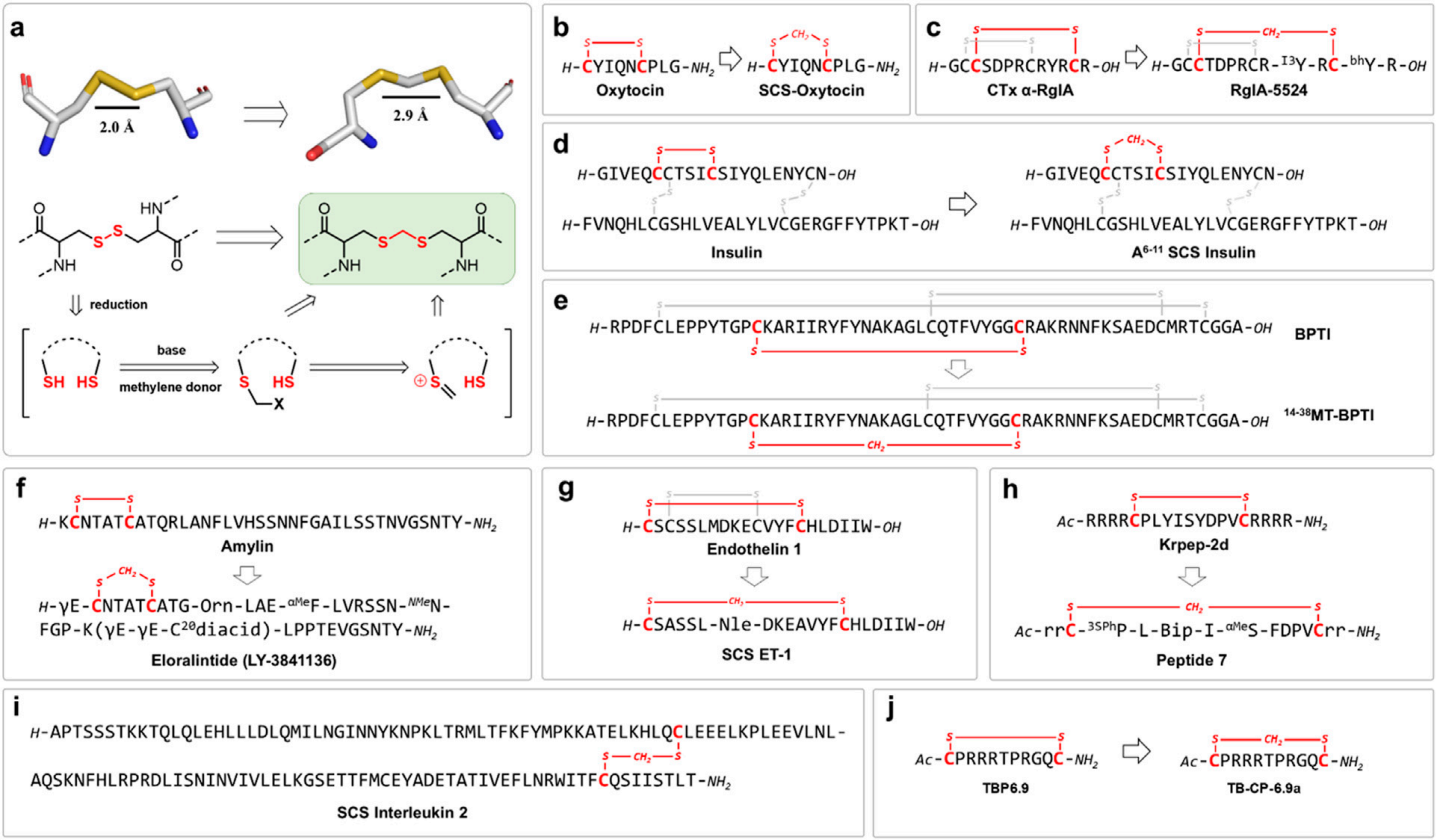
**(a)** Converting native disulfide into methylene thioacetal linkage by inserting the minimal “one-carbon” unit; Strategic applications of methylene thioacetal bonds as disulfide surrogates in bioactive peptides: **(b)** oxytocin; **(c)** conotoxin RgIA; **(d)** insulin; **(e)** BPTI; **(f)** human amylin; **(g)** human endothelin 1; **(h)** KRAS inhibitor; **(i)** interleukin 2 **(j)** HIV inhibitor.

Methylene thioacetal linkage has attracted growing interest for its favorable balance of chemical robustness, structural mimicry, and synthetic accessibility. In this strategy, the native disulfide bond is inserted with a minimal “one-carbon” unit CH_2_, preserving the spatial and conformational features of the original S-S bond while imparting resistance to both reductive cleavage and oxidative degradation. Several synthetic strategies have been developed to install the methylene thioacetal bonds, typically involving the selective reduction of native disulfides followed by alkylation of the resulting free thiols with methylene donors (Ueki et al., 1999; Kourra and Cramer, 2016). The most widely adopted method utilizes dihalomethane reagents, such as diiodomethane (CH_2_I_2_) and dibromomethane (CH_2_Br_2_), under mildly basic or neutral conditions. While methylene thioacetals are isosteric to disulfide bonds, subtle yet important differences in bond geometry and physicochemical properties influence peptide structure and function. Methylene thioacetals feature slightly longer S-S distance (2.9 Å) compared to native disulfide bonds (2.0 Å), and the additional methylene group introduces subtle changes to bond angles and local conformational preferences. This results in a marginally increased rigidity in certain peptide macrocycles and loops, which can be leveraged to enhance receptor binding affinity, proteolytic stability and plasma half-life (Figure 2a).

Investigations have revealed that methylene thioacetal substitution frequently preserves peptide folding and biological activity relative to their native disulfide-containing counterparts, with markedly enhanced serum stability and proteolytic resistance across a range of scaffolds, including conotoxins, anti-tumor peptides and hormone analogues. Notably, several methylene thioacetal modified lead compounds have advanced into clinical evaluation, underscoring the high translational potential of this strategy.

Cramer et al. introduced a mild, biocompatible one-pot methodology for converting native disulfide bonds in peptides into highly stable methylene thioacetal linkages (Kourra and Cramer, 2016). This straightforward and selective post-synthetic transformation occurs under aqueous conditions at ambient temperature, accommodating unprotected peptides with a range of functional groups. As a key application, the authors synthesized a methylene thioacetal-stapled analogue of oxytocin (SCS-OXT), which exhibited a dramatic enhancement in chemical and metabolic stability. In comparison to native oxytocin, SCS-OXT demonstrated complete resistance to reduction in glutathione-rich environments and significantly prolonged half-lives in human serum and at elevated temperatures. Notably, at physiological pH, SCS-OXT’s stability increased approximately 5-fold. Despite the minor structural modification, SCS-OXT retained full agonist activity at the oxytocin receptor, with a nanomolar EC_50_ in a uterine contraction assay (Figure 2b).

Conotoxins are a diverse family of small, disulfide-rich peptides derived from the venom of marine cone snails, evolved to target ion channels, receptors, and transporters with remarkable potency and selectivity (Jin et al., 2019). Their distinct ability to modulate neuronal signaling pathways has made conotoxins invaluable both as pharmacological tools and as leads for therapeutic development. However, the therapeutic exploitation of conotoxins faces significant challenges, chiefly due to their susceptibility to disulfide bond scrambling and oxidative instability under physiological and manufacturing conditions, which can compromise their structural integrity and bioactivity. As such, chemical modifications and alternative bond surrogates are increasingly recognized as necessary strategies to enhance their stability and broaden their clinical applicability. Chou et al. developed α-RgIA analogues incorporating methylene thioacetal bonds to enhance peptide stability and potency against human α9α10 nicotinic acetylcholine receptors (nAChRs), a promising non-opioid target for analgesic drug development (Zheng et al., 2021). The study revealed distinct regioselectivity in disulfide bond replacement: substitution of the disulfide in loop I (Cys^2^–Cys^8^) markedly impaired activity, whereas replacement in loop II (Cys^3^–Cys^12^) preserved potency. The lead analogue, RgIA-5524, incorporating a methylene thioacetal in loop II, demonstrated potent inhibition of human α9α10 nAChRs (IC_50_ = 0.9 nM), exceptional selectivity across a wide range of pain-associated ion channels and receptors, and significantly improved serum stability by preventing disulfide scrambling. Notably, *in vivo* studies showed that RgIA-5524 effectively prevented cold allodynia in a mouse model of oxaliplatin-induced neuropathic pain, an effect absent in α9 knockout mice, confirming robust target engagement. These findings position RgIA-5524 as a promising, stable, and selective α9α10 nAChR antagonist for neuropathic pain treatment. This work underscores how the strategic incorporation of methylene thioacetal surrogates can optimize the therapeutic potential of conotoxin-based, disulfide-rich peptides. However, replacing a disulfide bond in another conotoxin lead candidate Mr1.1 [S4Dap] with a methylene thioacetal, ether by loop I, loop II or both, resulted in complete loss of activity (Li et al., 2024b). This indicates that the use of methylene thioacetal as a disulfide bond surrogate still faces challenges in terms of selectivity and sequence compatibility, even in the same case of α-conotoxins (Figure 2c).

Insulin, a disulfide-rich peptide hormone, plays a crucial role in the regulation of blood glucose levels, with its biological activity and structural integrity critically dependent on three conserved disulfide bonds, including the A6-A11 linkage within the A-chain (Sims et al., 2021). However, insulin’s inherent instability, particularly its propensity to fibrillate and degrade under physiological and stress conditions, presents significant challenges for storage, handling, and therapeutic application (Richter et al., 2023). In response to these issues, Chou et al. engineered a human insulin analogue, SCS-Ins, by replacing the native A6-A11 disulfide bond with a methylene thioacetal surrogate (Zheng et al., 2019). Notably, SCS-Ins exhibited markedly improved resistance to fibrillation compared with native insulin, coupled with enhanced thermal and serum stability, showing superior resistance to degradation under both physiological and elevated temperature conditions. Importantly, SCS-Ins preserved its bioactivity, as confirmed by both *in vitro* assays and *in vivo* insulin tolerance tests in mice, where it exhibited comparable efficacy to native insulin. These results highlight the potential of methylene thioacetal incorporation at the A6-A11 position as a promising strategy to enhance the stability of insulin formulations. It is noteworthy that the SCS-Ins represents the limited examples of potent insulin analogues with A6-A11 disulfide replacement. Substitution of this interchain disulfide with either lactam, triazole bridge and dicarba bonds all led to potency deprivation (Figure 2d).

Understanding the process of protein folding is fundamental to deciphering how peptide chains acquire their functional structures and how misfolding can lead to disease (Hidaka and Shimamoto, 2013). Metanis et al. investigated the effect of substituting a native disulfide bond in bovine pancreatic trypsin inhibitor (BPTI) with the methylene thioacetal bridge. Remarkably, replacing the 14–38 disulfide bond preserved the native fold while revealing an alternative folding trajectory not observed in the wild-type protein (Mousa et al., 2018). This discovery highlights the subtle yet critical role individual disulfide bonds play in directing protein folding pathways and demonstrates that the methylene thioacetal substitution can maintain structural integrity while uncovering new mechanistic insights. The study underscores the value of methylene thioacetal as a versatile chemical tool for probing protein folding and stability (Figure 2e).

Amylin, a peptide hormone co-secreted with insulin by pancreas, plays a pivotal role in regulating glucose metabolism and appetite (Hay et al., 2015). The N-terminal disulfide bond in native amylin is crucial for its structural integrity and biological activity. However, amylin is highly prone to aggregation and fibrillation, particularly under physiological conditions, which limits its therapeutic potential (Wilkinson et al., 2023). Eloralintide (LY3841136), a long-acting amylin analogue, was developed to address these challenges, offering enhanced stability and prolonged action compared to native amylin. To improve its stability and mitigate aggregation, Eloralintide incorporates a methylene thioacetal surrogate in place of the native disulfide bonds. This modification significantly enhances resistance to fibrillation and improves its pharmacokinetic profile compared with native amylin. Currently, eloralintide is undergoing clinical evaluation in Phase 1 and Phase 2 trials for the treatment of obesity and overweight conditions, aiming to assess its safety, tolerability, and efficacy, both as a monotherapy and in combination with other agents such as tirzepatide (Figure 2f).

Endothelin-1 (ET-1) is a potent, 21-residue vasoconstrictive peptide that plays a key role in the regulation of vascular tone and cardiovascular homeostasis (Lankhorst et al., 2016). Its biological activity and structural integrity rely on two conserved disulfide bonds, which form a characteristic bicyclic scaffold essential for high-affinity receptor engagement. However, like many disulfide-rich peptides, ET-1 is prone to chemical instability and disulfide scrambling under physiological conditions, which can limit its therapeutic potential. In a recent study, a methylene thioacetal surrogate was introduced to replace one of the native disulfide bonds in ET-1, yielding a chemically stabilized single-loop analogue (Wolf and Beck-Sickinger, 2021). Notably, this modified peptide retained vasoconstrictor potency comparable to native ET-1, demonstrating that selective methylene thioacetal incorporation can preserve biological function while enhancing chemical stability (Figure 2g).

Beyond its established role as a disulfide bond surrogate in natural disulfide-containing peptides, methylene thioacetal has also been effectively applied to the optimization of bioactive macrocyclic peptides and lead compounds identified through library-based screening strategies. In a notable example, Heinis et al. reported the discovery of macrocyclic peptide inhibitors targeting KRAS, a historically challenging oncogenic protein implicated in a wide range of human cancers (Figure 2h). Through phage display-based selection and structure-guided optimization, the team incorporated methylene thioacetal bridges to constrain peptide conformation, significantly improving proteolytic stability while preserving high-affinity binding and enabling cell-active inhibition of KRAS signaling (Lim et al., 2021; Garrigou et al., 2022). Similarly, in efforts to target viral RNA structures, methylene thioacetal was employed in the design of macrocyclic peptide inhibitors against the HIV trans-activation response (TAR) RNA element (Chavali et al., 2020). Co-crystal structures of TAR RNA bound to the lead molecule TB-CP-6.9a revealed key arginine-mediated contacts essential for high-affinity binding, insights that guided the development of cyclic peptides stabilized with methylene thioacetal to maintain bioactive conformations while enhancing chemical and metabolic stability (Figure 2j). Together, these studies underscore the versatility of methylene thioacetal as a valuable tool not only for stabilizing natural peptides but also for advancing the development of structurally defined, pharmacologically robust macrocyclic peptide therapeutics.

Methylene thioacetal has emerged not only as a stabilizing modification for disulfide-rich peptides but also as a biocompatible surrogate for disulfide bonds within larger protein scaffolds. Bode et al. achieved the total chemical synthesis of interleukin-2, and the modified analogue incorporating a methylene thioacetal bridge in place of a native disulfide bond (Murar et al., 2020). Notably, the resulting analogue, compound 14, retained *in vitro* bioactivity comparable to the recombinant IL-2. This work highlights the broader applicability of methylene thioacetal in the chemical synthesis and stabilization of disulfide-containing proteins, while preserving their functional properties (Figure 2i).

## 5 Conclusion and outlook

Disulfide-containing peptides represent a valuable class of bioactive molecules with high target selectivity and potent pharmacological activities. However, the inherent instability of disulfide bonds, particularly their susceptibility to reduction and scrambling under physiological and manufacturing conditions, has long posed a barrier to their broader therapeutic application. Methylene thioacetal has emerged as a promising disulfide bond surrogate, offering a chemically robust, non-reducible alternative that preserves the native-like conformation and bioactivity of peptide scaffolds. However, despite these advances, challenges remain regarding sequence-specific compatibility and potential effects on target binding and functional activity.

Moving forward, comprehensive *in vivo* studies addressing immunogenicity, toxicity, and pharmacodynamics, developing predictive guidelines for sequence compatibility, and integrating these chemistries into modern peptide display and screening platforms will be essential to fully realize the therapeutic potential of this promising strategy. In this landscape, methylene thioacetal stands as a powerful and versatile tool not only for stabilizing natural peptides but also for enabling the next-generation of chemically resilient, bioactive macrocycles in peptide drug discovery.
